# Tracking physical activity using smart phone apps: assessing the ability of a current app and systematically collecting patient recommendations for future development

**DOI:** 10.1186/s12911-020-1025-3

**Published:** 2020-02-03

**Authors:** J. Murphy, T. Uttamlal, K. A. Schmidtke, I. Vlaev, D. Taylor, M. Ahmad, S. Alsters, P. Purkayastha, S. Scholtz, R. Ramezani, A. R. Ahmed, H. Chahal, A. Darzi, A. I. F. Blakemore

**Affiliations:** 1 Department of Surgery, Cancer and Investigative Medicine, Imperial College London, London, UK; 2 Warwick Business School, University of Warwick, Coventry, UK; 3 Psychology Department, Manchester Metropolitan University, Manchester, UK; 4 Behavioural Science Group, Warwick Business School, University of Warwick, Coventry, UK; 5 Big Data Analytical Unit, Imperial College London, London, UK; 6 Section of Investigative Medicine, Division of Diabetes, Endocrinology, and Metabolism, Department of Medicine, Imperial College London, London, UK; 7 Imperial Weight Centre, Imperial College Healthcare NHS Trust, St. Mary’s Hospital, London, UK; 8 Wireless Health Institute, University of California, Los Angeles, USA; 9 Department of Life Sciences, Brunel University London, London, UK

**Keywords:** Obesity, Physical activity monitoring, Smartphone app, Surgery

## Abstract

****Background**:**

Within the United Kingdom’s National Health System (NHS), patients suffering from obesity may be provided with bariatric surgery. After receiving surgery many of these patients require further support to continue to lose more weight or to maintain a healthy weight. Remotely monitoring such patients’ physical activity and other health-related variables could provide healthworkers with a more ‘ecologically valid’ picture of these patients’ behaviours to then provide more personalised support. The current study assesses the feasibility of two smartphone apps to do so. In addition, the study looks at the barriers and facilitators patients experience to using these apps effectively.

****Methods**:**

Participants with a BMI > 35 kg/m^2^ being considered for and who had previously undergone bariatric surgery were recruited. Participants were asked to install two mobile phone apps. The ‘Moves’ app automatically tracked participants’ physical activity and the ‘WLCompanion’ app prompted participants to set goals and input other health-related information. Then, to learn about participants’ facilitators and barriers to using the apps, some participants were asked to complete a survey informed by the Theoretical Domains Framework. The data were analysed using regressions and descriptive statistics.

****Results**:**

Of the 494 participants originally enrolled, 274 participants data were included in the analyses about their activity pre- and/or post-bariatric surgery (ages 18–65, M = 44.02, SD ± 11.29). Further analyses were performed on those 36 participants whose activity was tracked both pre- and post-surgery. Participants’ activity levels pre- and post-surgery did not differ. In addition, 54 participants’ survey responses suggested that the main facilitator to their continued use of the Moves app was its automatic nature, and the main barrier was its battery drain.

**Conclusions:**

The current study tracked physical activity in patients considered for and who had previously undergone bariatric surgery. The results should be interpreted with caution because of the small number of participants whose data meet the inclusion criteria and the barriers participants encountered to using the apps. Future studies should take note of the barriers to develop more user-friendly apps.

**Trial registration:**

ClinicalTrials.gov- NCT01365416 on the 3rd of June 2011.

## Background

The prevalence of obesity among adults in the United Kingdom increased from 14.9% in 1993 to 25.6% in 2014 [1]. To help people lose weight, interventionists within the National Health Service (NHS) can encourage them to change their lifestyle, provide them with medications, and, if suitable, provide them with bariatric surgery [2]. Bariatric surgery is currently the most effective long-term treatment for severe obesity (particularly in the presence of Type 2 diabetes). However, its cost-efficacy for uncomplicated obesity is debated [3], and even after receiving bariatric surgery 10 to 20% of patients still experience suboptimal long-term weight loss [4, 5]. The long-term success of bariatric surgery depends in part on patients’ adherence to physical activity recommendations [6–9], and many patients likely require additional support to follow through on their good intentions. The current article seeks to evaluate the feasibility of using smartphone apps to track such patients’ physical activity and other health-related variables.

### Physical activity

Public health guidelines from the United States and United Kingdom recommend that adults engage in at least 150 min per week of moderate-to-vigorous physical activity (MVPA) and minimise sedentary behaviour to enhance health-related outcomes [10]. These guidelines extend to patients with chronic conditions or disabilities where they are able to engage. After undergoing bariatric surgery patients are encouraged to engage in MVPA for at least 10-min bouts every day [11, 12]. A 2016 review of 50 studies measuring such patients’ physical activity suggests that many patients do increase their physical activity post-bariatric surgery [13]. However, only 7 of the 50 studies included objective measures of physical activity, so the inference that these patients’ physical activity increased largely relies on self-reports that may be affected by response biases, e.g. social desirability. A further concern is that of these seven studies only one assessed patients’ MVPA [14]. This study suggests that 89% of patients do not engage in the recommended MVPA in at least 10-min bouts every day. So even patients who increase their physical activity may still not be engaging for sufficient durations and or at sufficient intensities to gain the optimal benefit. Whilst historically tracking physical activity in real-time has been difficult, newer activity-monitoring tools are increasingly capable.

### Monitoring physical activity using technology

Whilst offering patients support to increase their physical activity is encouraged by NHS England’s Obesity Clinical Reference Group guidelines, such support is often lacking in part because practitioners do not know how active patients are [15]. Providing patients with activity-monitoring tools could help practitioners offer support in a data-led fashion. This possibility encourages the development of technologies that can obtain richer and more objective physical activity profiles, such as smartphone applications (hereafter referred to as ‘apps’). Pew Research Centre’s (2019) research suggests that 76% of the UK adults currently own a smartphone [16], and a number of apps already exists that allow users to track their daily physical activity and create physical activity profiles. A number of recent studies have already used apps to track weight loss [17–19]. Ross and Wing’s study included an analysis of 68 million days of physical activity for over 700,000 people worldwide, and found that people who were less active were more likely to be obese [20].

A reliable app that tracks physical activity could eventually be used to deliver timely interventions to patients. These apps should be ergonomically designed and motivate users to be more active. For example, patients who do not engage in sufficient activity for multiple days could be sent a text-message that motivates them to move more or to schedule an appointment. The apps may even help patients motivate themselves by prompting them to set daily, weekly, and monthly targets, as well as by sending them automated reminders to engage in exercise based on their self-set goals. For example, a motivating personalised message might say, “[Name], you are almost there, an 8 minute brisk walk will allow you to achieve your move goal today”.

From a research perspective, the importance of developing mobile health (i.e. mHealth) technologies that are user-friendly and easily accessible is important in order to encourage participants to record their data. From a practice perspective, reliable apps that track and motivate patients physical activity could help practitioners increase what are typically poor following-up rates with patients after they have had bariatric surgery [21]. Many patients miss in-person follow-up appointments for various reasons, such as the distance they need to travel or their ability to get time off work [22]. Enabling these patients to attend their follow-up appointment remotely, informed by their own physical activity data, may be sufficient to increase the long-term success rates of bariatric surgery [23–25]. There are already many apps for patients’ with chronic health conditions and many users have positive reactions to them, e.g. they experience an increased sense of agency [26]. Thomas et al. argue that these technologies are not only cost-effective but are also ecological valid feedback tools [27]. As highlighted by Bradley et al.’s review, it appears that patients are receptive to remote assessment [28]. Despite these advancements in app technologies, the feasibility of remotely monitoring patients’ health-related data via apps is largely unexplored, few studies address evidence-based physical activity guidelines, and there is greater scope for these apps to deliver theoretically informed and empirically supported behaviour change techniques [29].

### The current study

The current study aims to evaluate the feasibility of smartphone apps to monitor patients’ physical activity pre- and post-bariatric surgery. Whilst physical activity is the core focus of the current study, other health-related data were also considered, including weight, mood, wakefulness, and satisfaction. These are important health-related variables that impact on physical activity [26, 30–32] and considering them here may help inform the development of future apps. Notably, the reliability of apps depends on more than the technology itself. Specifically, the apps’ reliability also depends on patients using the technology appropriately, e.g. having their phone switched on and kept on their person. To capture this aspect of the apps’ reliability several participants in our study also completed surveys informed by the Theoretical Domains Framework (TDF). The TDF captures the behavioural facilitators and barriers per theoretically and empirically informed behaviour change domains [33]. The information gathered in the current TDF survey can be used to identify common barriers that future apps should overcome to improve their usability.

## Methods

The current study formed part a clinical trial registered at Clinical Trials.gov (ID: NCT01365416) on the 3rd of June 2011. This study was given favourable opinion to conduct by The London-Riverside research ethics committee (Reference: 11\LO\0935). Recruitment took place between September 2014 and July 2015. Participants could use the apps for as long as they wished, though their activity was only monitored whilst the study was active. The methods section now reviews the current study’s aims, participants, and measures.

### Aims

The current study has four aims:

1. Remotely monitor patients’ physical activity pre- and/or post-bariatric surgery via the Moves app.
2. Remotely monitor the same patients’ other health-related variables (weight, mood, wakefulness, and satisfaction) via a weight loss companion app, called the WLCompanion app.
3. Assess the relationship between physical activity and other health-related variables using regression analyses.
4. Identify the main facilitators and barriers patients experience using the apps via a TDF informed survey.

### Participants

To be eligible for the study patients had to be ambulatory, be 18–65 years old, have a body mass index > 35 kg/m^2^, have access to an Apple or Android smartphone, and either be considered for bariatric surgery or had already undergone their surgery (including gastric bypass, band or sleeve). Patients likely to be eligible were approached and recruited at outpatient appointments in the Imperial Weight Centre, St. Mary’s Hospital, London. Recruitment started in September 2014 and participants were at different stages of their weight loss journey.

**Fig. 1.**
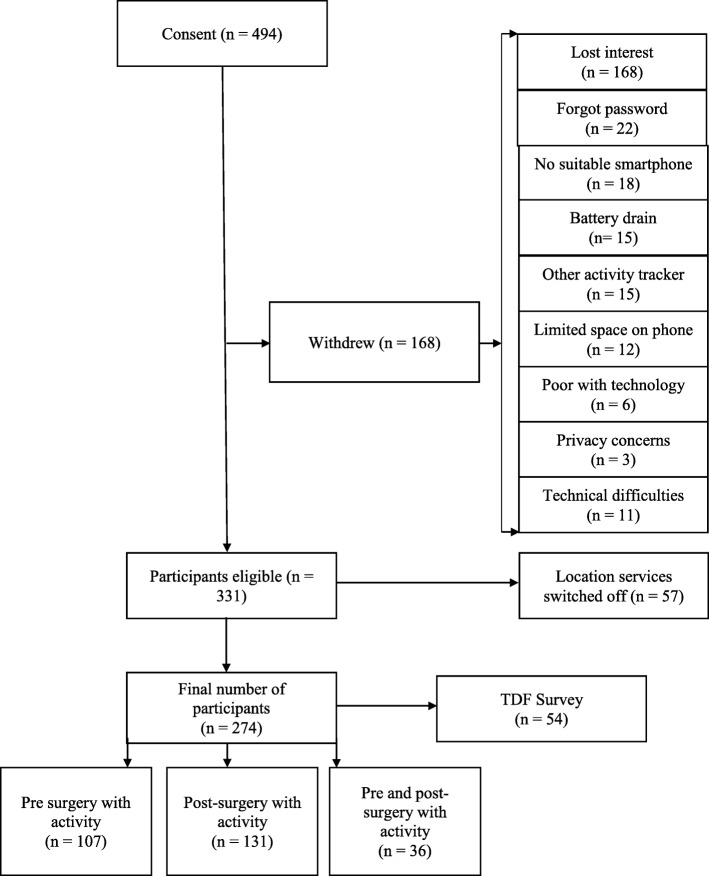
Flow of participants through the study

As part of the recruitment participants who consented to take part agreed to the apps’ terms and conditions, and gave the research team permission to access their anonymised data. Participants were provided with a support link that directed them to a website to read about the progress of the research programme and enabled them to send individual feedback to the research team. Participants were asked at each of their follow-up clinic visits if they wished to continue participating in the study and could withdraw at any time and without giving a reason and without it affecting their treatment. A subset of the participants recruited opportunistically were also asked to complete a TDF survey at Imperial Weight Centre. The surveys were completed on an iPad using Qualtrics software. Fig. 1 shows the flow diagram of participants in the study. Demographic information about the participants is provided in Table 1.

**Table 1:** Demographic and clinical characteristics of the patient cohort

Baseline Characteristics	Of 274 participants with sufficient App data	Of the 54 participants who completed the TDF survey
Age (M, SD)	44.0 years, SD = 11.14	42 years, SD = 9.7
Gender		
Female	161, 67.3% retention	39
Male	113, 44.4% retention	15
Ethnicity		
White British	44.5%	46.2%
White Other	14.2%	12.9%
Indian	5.1%	5.5%
Pakistani	1.8%	1.8%
Other Asian	1.5%	1.8%
Caribbean	8.8%	9.2%
African	4.7%	3.7%
Any Other	12.0%	12.9%
Mixed	7.3%	5.5%
Preoperative BMI Mean (SD)	46.5 BMI SD = 8.6	49.4BMI, SD = 9.5
Preoperative BMI Range		
< 35 kg/m^2^	8.0%	7.5%
35-40 kg/m^2^	15.3%	13.4%
40-50 kg/m^2^	46.4%	43.2%
> 50 kg/m^2^	26.3%	34.5%
Type of Surgery		
Pre-Surgery	28.8%	31.1%
Roux-en-Y Gastric Bypass	38.3%	34.7%
Sleeve Gastrectomy	23.0%	25.7%
Gastric Band	5.8%	4.5%
Revision	4.0%	3.6%
Pre-Surgery Functional Status		
Requires Walking Aid	11.0%	11.3%
Can manage 1 or 2 flights of stairs	43.6%	46.5%
Can manage 3 flights of stairs	23.4%	22.3%
No limitation	22.0%	19.6%

### Measures collected by apps

To access the two apps participants were given a link to download them on their smartphones. All data collected were anonymised using numerical codes. The core purpose surrounding the apps was to minimise the direct level of human involvement: By having the physical activity tracking app installed on participants’ mobile phone, data collection about their physical activity did not require the users to do any more than passively carry their phones with them as they carried out their daily activities. The companion app sent participants prompts to encourage physical activity and reminders to manually input their weight and other health-related data. Participants could personalise what information they wished to record and how often they wanted to be sent reminders to do so by altering their settings directly on the app. Participants were also encouraged to download and share their data with their general practitioners, friends, or medical specialists to evidence their progress and discuss how they may be getting on.

#### Moves app: measurement of physical activity

Participants’ physical activity was automatically recorded via their smartphones, using an app called Moves, developed by ProtoGeo. Moves was selected because it was one of the most widely-used free apps with an acceptable interface to gather experimental data. Further information about this app is provided in Additional File 1. The physical activity recorded on the app included walking, cycling, and running, and through the app participants were able to view the distance, duration, steps, and an estimate of calories burned for each of these activities. Moves measures users’ physical activity via the phones’ inbuilt accelerometer and global positioning system. Moves runs in a phones’ background and transmits data to a server when the phone has an internet connection.

For participants’ daily Moves data to be included in the final analysis, their smartphone’s location services had to be switched on 50% of the time between 6:00 and 22:00 for at least one day. This criterion should not suggest that one day is a sufficient amount of time to measure a person’s common daily physical activity, rather these data were included in the current study because they help understand the feasibility of participants using the app in a care capacity, e.g. ecological validity. The recorded physical activity was categorised for our analyses as follows: 1) average walking time per day, 2) average time spent walking at > 80 steps/min, i.e. MVPA, and 3) average number of MVPA in ≥10-min bouts of activity per day.

#### WLCompanion app: measurement of weight, mood, wakefulness, and satisfaction

Other health-related data were recorded via participants’ smartphones using a companion app called WLCompanion developed by Imperial College London. A screen shot of this app is provided in Additional File 1. WLCompanion reminded participants to input their weight in kilograms (or stones and pounds), and to rate their mood, wakefulness, and satisfaction on five-point Likert scales. Participants choose whether they wanted to be reminded to input these data daily or weekly. Participants were also able to record additional health-related activities that the Moves app could not, e.g. swimming. Additionally, the WLCompanion app presented summative information on participants’ progress based on the data from Moves and WLCompanion. Participants and the research team could see this summative information.

#### Behaviour change technique

The apps described above could prompt behaviour changes related to weight loss. Drawing on the work of Michie, Atkins and West [34], the behavioural change techniques used in the current study are as follows: goals and planning; feedback and monitoring; and associations. Regarding the goals and planning technique, participants were able to *set weekly goals* about their physical activity. Regarding the feedback and monitoring technique, participants were able to *self-monitor* their mood and satisfaction with their weight loss, and received *feedback on outcomes of behaviour* as summative reports. Finally, regarding the associations technique, participants received *prompts* that included weekly weigh-in reminders and messages telling them to engage in more exercise if they were falling behind. These behaviour change techniques are interlinked with the TDF survey.

### TDF survey

The TDF informed survey was designed to capture facilitators and barriers participants experienced to using the apps [33]. The TDF is an important tool for improving the implementation of evidence-based practice, and allows research teams to consider additional factors that may influence behaviour. The TDF is a widely used tool in a range of healthcare and behaviour change settings [35] and has been reported to be a valid framework around which to develop inventories [33]. The TDF consists of 14 domains of which 13 were measured in this project; the ‘Optimism’ domain was excluded as it overlapped too much conceptually with the ‘Beliefs in Consequences’ domain. Each domain was assessed with 3 to 11 items. Each item was presented as a statement, and participants indicated their agreement with that statement on a five point Likert scale. For example, an item designed to assess the ‘Environmental Context and Resources’ domain read: “I always keep my phone charged” [response options range from 1 = strongly disagree and 5 = strongly agree]. The survey’s items appear in in Additional File 2.

### Data analysis

To assess and establish relationships in relation to the health tracking tools, a series of statistical analyses were conducted. First, linear regression analyses were conducted to examine the relationship between physical activity and other health-related variables (mood, wakefulness, the interaction between mood and wakefulness, satisfaction, age, and surgery stage). In lieu of the small samples sizes, these results should be interpreted in an exploratory capacity.

Second, in order to identify the facilitators and barriers to app use, which is considered important in the uptake of mobile apps for remotely monitoring physical activity, participants responses to the TDF informed survey were descriptively examined. The data included 54 participants. Of these participants 11 were at a pre-surgery stage of their journey, and so completed items related to their post-surgery intentions but not their post-surgery behaviours. To examine participants’ responses, each participant’s 13 domain scores were obtained by calculating each participant’s mean responses to the items within each domain. Then the overall participants’ domain scores were obtained by calculating the median participant domain scores for each of the 13 domains, along with the 25th and 75th percentiles. Participants’ responses were coded such that lower scores indicated a greater barrier to their physical activity.

## Results

### Remotely monitoring physical activity

Regarding Moves, 274 participants’ activity was tracked for a median of 131 days, (range = 1–420, IQR = 36–148). Table 2 describes the number of days participants’ Moves data were recorded. Note that seven participants had their activity recorded for only one day. The huge variability in the number of days of physical activity recorded for participants was in part due to factors such as their turning off mobile location services needed for the app to collect data or deleting the app and downloading it again at a later date. The analyses below are split by the stages in which participants’ activity was tracked: pre-surgery, post-surgery or from pre- to post-surgery.

**Table 2:** The number of days participants’ data were recorded

Days recorded	Number of participants
1 to 10	35
11 to 20	19
21 to 40	29
41 to 60	31
61 to 80	21
81 to 100	21
101 or more	118

#### Pre-surgery

Of the 274 participants, 107 were tracked only pre-surgery for a median of 62 days (range = 1–245, IQR = 27–119). As measured by Moves, the median number of steps per day was 1130 (range = 195–4345, IQR = 536.5–1773), and participants walked a median of 15.48 min per day (range = 2.67–48.13, IQR = 7.27–21.18). Only 18.6% of participants achieved at least 30 min of activity daily. Whilst 46.5% of participants engaged in MVPA, these participants only did so for a median of 5.81 min per day (range = 0.30–108.6, IQR = 0.58–4.21). Only 28.5% of participants engaged in at least 1 bout of MVPA lasting ≥10 min per day; of these participants their median bout was 19.12 min (range = 11.5–32.52, IQR = 15–22.01).

#### Post-surgery

Of the 274 participants, 131 were tracked only post-surgery for a median of 67 days (range = 1–245, IQR = 32–133). As measured by Moves, participants walked a median of 19.57 min per day (range = 2.25–72.74, IQR = 10.07–27.53). The median number of steps per day was 1460 (range = 138–4159, IQR = 753–2391). Only 24.3% of participants achieved at least 30 min of activity daily. Whilst 73.5% of participants engaged in MVPA, they only did so for a median of 12.05 min per day (range = 0.14–70, IQR = 0.35–5.57). Only 39.5% of participants engaged in at least 1 bout of MVPA lasting ≥10 min per day; of these participants their median bout was 27.21 min (range = 10.01–60, IQR = 13.55–35.48).

#### Pre-surgery to post-surgery

**Fig. 2.**
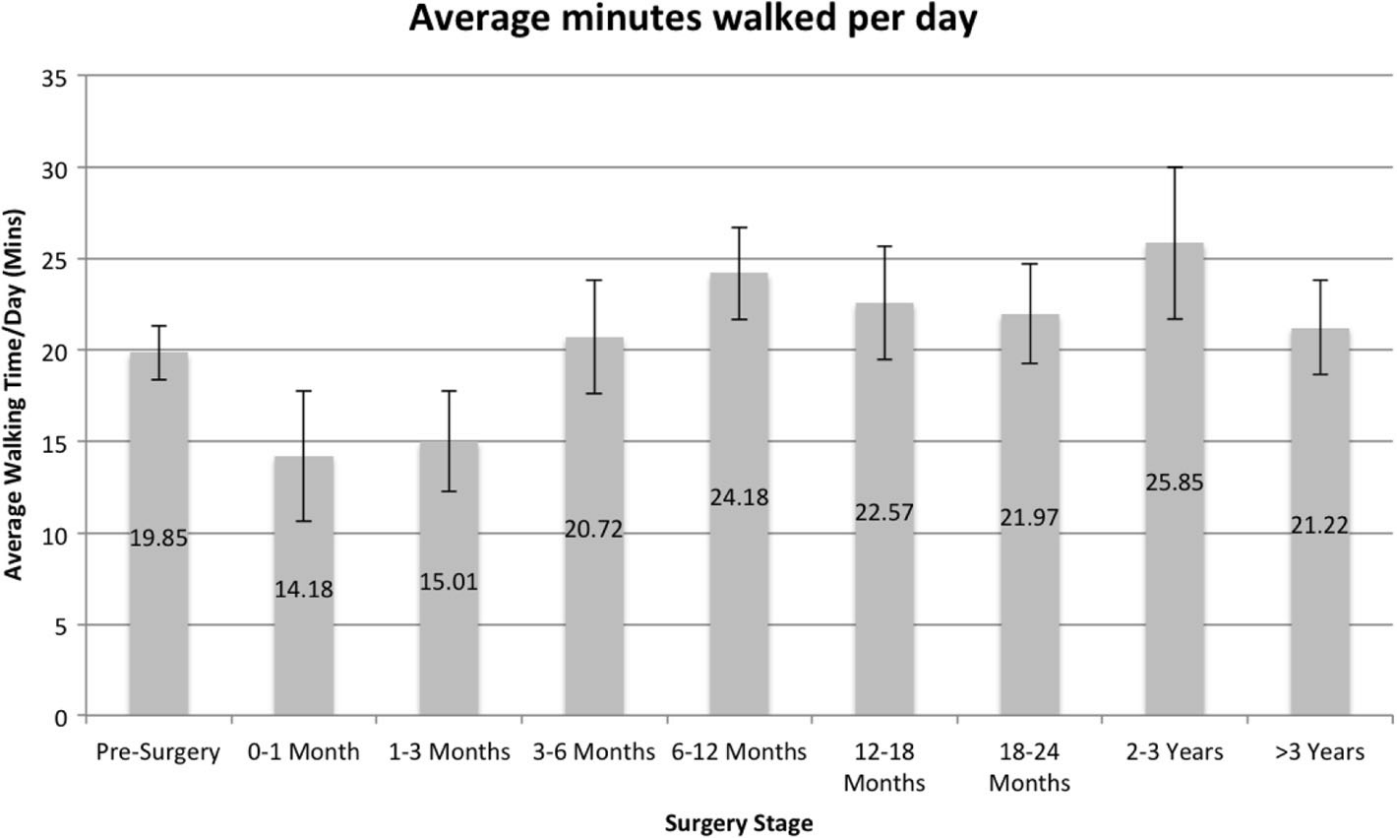
Daily average walking time from pre-surgery to >3-years post-surgery (Error bars = 1 Standard Error)

The remaining 36 participants were tracked from pre-surgery for a median of 51 days (range = 2–117, IQR = 23–63) through post-surgery for a median of 62 days (range = 11–176, IQR = 39.25–124.5). The participants’ daily activity levels were divided into groups based on how much time had passed since their surgery and visually examined for trends. Fig. 2’s left most bar represents participants’ daily mean walking times pre-surgery, followed by bars representing participants’ daily average walking times for periods post-surgery. Compared to pre-surgery activity levels, initially post-surgery activity decrease. However, by months three to six, post-surgery activity levels roughly resume their pre-surgery levels and then stabilise.

### Remotely monitoring other health-related variables

Regarding WLCompanion, 117 participants entered their weight, mood, wakefulness, and satisfaction 12 days on average (Mdn = 4). Of these participants 46 used the apps only pre-surgery, 37 used the apps only post-surgery, and 34 used the apps both pre- and post-surgery.

### Assessing the relationship between physical activity and other health-related variables

Linear regressions were performed to assess whether the data recorded on WLCompanion predicted different characteristics of participants’ physical activity. Specifically, the following variables were entered as predictors: mood, wakefulness, the interaction between mood and wakefulness, satisfaction, age, and surgery-stage (pre- or post-surgery) to predict participants’ speed, steps per day, and duration of activity. Due to the small sample size, these analyses should be considered exploratory, and their results interpreted in an exploratory capacity.

#### Speed

Regarding speed, the results of the regression indicated that mood was the only significant predictor. Mood explained 7% of the variance in speed (R^2^ = 0.07, F(89, 1938) = 1.74, *p* < 0.001; mood B = 0.07, *p* = 0.04). This indicates that participants with more positive moods tended to move faster.

#### Steps per day and duration of activity

Regarding steps per day and duration of activity, the results of the regressions were more nuanced. The same three predictors significantly contributed to the model for steps per day (R^2^ = 0.32, F(89, 1939) = 10.22, p < 0.001) and duration of activity (R^2^ = 0.32, F(89, 1939) = 10.98, p < 0.001). Regarding steps per day, the amount each predictor contributed was as follows: mood (B = 0.19, *p* = 0.001), satisfaction (B = − 0.10, p = 0.001), and age (B = − 0.92, p < 0.001). Regarding duration of activity, the amount each predictor contributed was as follows: mood (B = 0.19, p = 0.001), satisfaction (B = − 0.14, p < 0.001), and age (B = − 0.77, p < 0.001). Thus, whilst a good mood positively contributed to participants’ steps per day and duration of activity, participants’ satisfaction with their weight loss journey and age negatively contributed.

### Identifying the facilitators and barriers to the apps use

The data from the 54 participants who completed the TDF survey were analysed. Of these participants 11 were pre-surgery and so only completed the items related to their post-surgery intentions. The overall participants’ median and percentile scores are provided in Table 3. As a reminder lower scores indicate that participants experienced such domains as greater barriers to physical activity. The domains with the lowest scores included ‘Environmental Context and Resources’ (Mdn = 2.95), followed closely by ‘Beliefs about Capabilities’ (Mdn = 3.00), and ‘Emotions’ (Mdn = 3.11). The domains with the highest scores included ‘Intentions’ (Mdn = 5.00), ‘Belief about Consequences’ (Mdn = 4.00), and ‘Social Identity’ (Mdn = 4.00).

**Table 3:** Participants’ median responses to each theoretical domain

Theoretical Domain	Number of Responders	Overall Domain Score Median	25th Percentile	75th Percentile
Environmental Context & Resources	54	2.95	2.82	3.27
Belief about Capabilities	54	3.00	2.33	3.67
Emotion	54	3.11	2.89	3.33
Memory Attention & Decision Making	54	3.30	2.80	3.60
Goals	54	3.33	3.00	3.67
Behavioural Regulation	54	3.40	3.00	4.00
Reinforcement (automaticity)	54	3.50	3.00	4.00
Knowledge	54	3.63	3.38	3.88
Social Influences	54	3.67	3.00	4.00
Skills (cognitive)	54	3.75	3.25	4.00
Social Identity	54	4.00	3.67	4.33
Belief about Consequences	54	4.00	3.67	4.33
Intentions (post-surgery)	11	5.00	4.00	5.00

## Discussion

### Principle findings

Overall, the current study addressed four main aims to assess the feasibility of incorporating technologies through smartphones to track physical activity and other health-related behaviours in a clinical population. The first aim was to remotely monitor patients’ physical activity pre- and post-bariatric surgery. Notably, the current study found that patients’ physical activity did not change from pre- to post-surgery, and most patients did not engage in sufficient MVPA. These results are similar to previous findings measuring physical activity recorded via accelerometers and questionnaires [36]. The second aim was to measure other health-related variables via a companion app. The companion app allowed data to be collected on weight, mood, wakefulness, and satisfaction; however, patients likely need further incentives to manually input such information more frequently. Whilst participants may find it easier to use a single app, the current research team cautions interventionists to avoid what Norman calls ‘featuritis’; a temptation to add more features to a single app that will ultimately weaken the app’s usability [37].

The third aim was to assess the relationship between physical activity and other health-related data. Mood was the most reliable predictor of participants’ physical activity: participants with more positive moods tended to engage in more physical activity. This finding is consistent with previous research. For example some researchers have found that helping people form positive expectations about exercising increases the enjoyment they get from exercising and their intentions to engage in it [38]. Just how interventionists can trigger such positive expectations is an exciting area for future research.

The fourth aim was to assess the facilitators and barriers people experience to using apps to monitor physical activity and other health-related data. User feedback on the usability of Moves and WLCompanion suggests that participants were more likely to use the apps when the information was recorded automatically. Unfortunately, automatic recording (and use of phones’ location services) increases the rate of a smartphone battery draining. This caused the research team to miss a lot of potentially fruitful data. A recent study by Orr et al. compared various smartphone pedometer applications (i.e. Accupedo, Moves, and Runtastic pedometer) and found an unacceptable rate of accuracy in all the applications compared to a handheld pedometer [39]. This does not mean that these apps are not useful, rather it suggests that these apps stand to be improved.

Overall, the current study’s aims are now explored in relation the developing mHealth technologies, particularly moving towards collecting data in real-time to get more accurate and ecologically valid data to inform research and clinical practice. As previous research has found that patients who experience bariatric-surgery patients are receptive to remote assessments, it is important view the current findings as a foundation for future research utilising mHealth interventions. As patients seem to have difficulty attending in-person follow-up appointments after bariatric surgery, remote follow-up appointments informed by real-time data may be necessary to help more experience successful long-term weight loss [40, 41]. The findings from the current study, shed some light on how apps could be used more effectively, as well as understanding how things like mood may affect physical activity.

### Strengths

The current study has a number of strengths. Firstly, it included an evaluation of real-time data rather than relying on subjective self-report measures. The study was able to record speed, steps per day, and duration of activity. The population selected for the study was important as it allowed for comparisons pre- and post-surgery, where post-surgery, weight maintenance through physical activity is key. Interestingly, no statistically significant changes in physical activity were evident from pre- to post-surgery and therefore questions arise as to what other factors or interventions are needed to promote physical activity, a promising line of enquiry where apps could play a significant role.

A further strength of the study was its inclusion of a TDF informed surveys to evaluate the facilitators and barriers to using the apps. The findings here suggest which barriers future interventions should help patients overcome. For example, as the ‘Beliefs about Capabilities’ domain was one of the largest barriers to physical activity, and so future interventions may focus on understanding/enhancing patients’ beliefs about their capabilities, i.e. self-efficacy.

### Limitations

A number of limitations are discussed now. First, it is important to acknowledge the high attrition rate. Specifically of the 494 participants recruited, only 272 (55%) had sufficient data to be included in our analyses. This limitation negatively impacts the certainty of our findings. However, this limitation is itself an interesting finding. The difficulty participants experienced installing or using the mobile apps caused much of this attrition. Additionally, the two apps did not work together as seamlessly as possible. Indeed, Bradley et al.’s work suggests that bariatric patients are often receptive to remote assessment and interventions [28], but the success of those interventions likely depends on the apps being easy for patients to use. People often lose interest in an app after the first month of a study [42]. Less time-consuming and more engaging apps could produce better weight loss outcomes [43]. As a reminder, the current study’s inclusion criteria liberally including participants who only had 1 day’s data. Future studies may set more conservative criteria for data inclusion.

Several participants reported that they did not tend to keep their phone on their person, and so short walking trips were often not recorded. Advances in technology have progressed with wearable fitness trackers that may pick up more data in future trials. For example, Wang et al. [44] examined the use of fitness trackers over 6 weeks and found that only a small increase in MVPA. Finally, regarding WLCompanion, the median participant entered their weight, mood, wakefulness, and satisfaction on only 4 days. This was notably fewer days than the Moves app that automatically collected participants’ data (Mdn = 131 days). This finding underscores the importance of automatically collecting data when possible.

### Implications

The current study has implications for the development of new technologies and for patients undergoing bariatric surgery. Regarding the development of new technologies, it is important to note that the data were collected from 2014 to 2015 and technology has since progressed. At the time of this study, physical activity tracking apps were unique, novel, and arguably still in the development phases. Currently, in 2019, many smartphones regularly include more advanced physical activity tracking apps. Whilst the technology around physical activity tracking has certainly advanced, the combined use of this technology with behaviour change techniques lags behind. One features that makes the current study still relevant is its integration of a companion app through which behaviour change techniques could be delivered in real-time based on patients’ real-world activity. The barriers and facilitators found in this study still apply to the new technologies being developed.

Regarding patients undergoing bariatric surgery, the current study suggests that further education is needed to reiterate the importance of physical activity in order to maintain a healthy lifestyle. Whilst this study focused on bariatric surgery patients, its findings may be useful in other clinical relevant behaviours. For example, Zhang et al. used mobile technologies with a bio-feedback loop to help patients diagnosed with anorexia nervosa overcome their compulsive need to exercise [45].

### Recommendations

There are a number of recommendations that could be considered for a range of studies using technology to collect data. Although the current study focused on a specific cohort of participants, these recommendations are not limited to bariatric-surgical patients. Critically, the current study did not aim to assess how seasonality influences participants’ movement, future studies may include seasonality as an additional factor in their analyses. Future studies may look at ways to automatically collect such information or to motivate patients to manual input such information more reliably. Moreover, as we have acknowledged, technology has advanced since this study commenced, we do, however, recommend future apps should be easier to install. The findings from our study suggest other ways future apps can be improved to minimise attrition rates, in practice and research.

## Conclusion

In conclusion, our results suggest that patients’ physical activity does not change significantly after bariatric surgery, and that their MVPA often remains below recommended levels. Patients’ lack of physical activity post-bariatric surgery is one of several reasons why 10–20% of patients fail to achieve optimal weight loss after receiving bariatric surgery [4]. Developing reliable, low-cost, and non-invasive technology to help remotely monitor patients’ physical activity could enable practitioners to support patients who are not active enough precisely when they need that help, e.g. they could send inactive patients an automated text-message or letter inviting them to come to existing support groups. Whilst smartphone app technology has not reached that point of development yet, the current study puts forth advice to improve the usability of future apps.

## Supplementary information

**Additional file 1.** Information about how and what data the apps collected

**Additional file 2.** Theoretical Domains Framework Survey Items

## Data Availability

The datasets used and/or analysed during the current study are available from the Jennifer Murphy at j.murphy@imperial.ac.uk on reasonable request.

## Abbreviations

MVPA: Moderate-to-vigorous physical activity
TDF: Theoretical Domains Framework
