# Supplementary material for: Tracking physical activity using smart phone apps: assessing the ability of a current app and systematically collecting patient recommendations for future development
Source: BMC Med Inform Decis Mak. 2020 Feb 3;20:17. doi: 10.1186/s12911-020-1025-3 (PMC6998214; doi:10.1186/s12911-020-1025-3)
Supplement: Supplementary file 1 — Additional file 1. Information about how and what data the apps collected [file 12911_2020_1025_MOESM1_ESM.docx]

**Additional File 1.**

Supplementary Information. The Apps: Moves and WLCompanion

**The Moves App**

The Moves app by ProtoGeo can automatically records any walking, cycling, and running the user does while carrying their smartphone. Users can view the distance, duration, steps, and an estimate of calories burned for each activity. The app is always on, so there is no need to start and stop it. It uses the smartphone’s inbuilt sensors which is advantageous for research purposes as it does not require the user to do any more than passively carry their phones as they carry out their daily activities. There is no need to charge, remember to wear, or carry another item, and hence the user’s activity data can be collected more comprehensively and over much longer periods of time.

**The WLCompanion App**

This app provides the user with weigh-in reminders and also enables them to record their mood, overall satisfaction with their weight loss and to log any additional exercise (such as swimming) that they have done without their phone. WLCompanion gives the user choices over which data to collect and the frequency (either daily or weekly). In addition, users can set a weekly goal for physical activity and choose to be reminded to take a walk if they are falling behind.

The following text displays information that can be provided on a website and when the WLCompanion app first runs:

*Weight loss Companion will help you to keep track of your weight loss journey and your feelings. When used together with the Moves app it will provide you with a daily or weekly record of your mood, physical activity and weight. We hope that this will help you to set sensible goals for yourself and to see how you are progressing against those goals. Please discuss your goals with your medical advisors to ensure that they are realistic and appropriate for your personal circumstances.*

*We will use data from the Weight loss Companion app (in combination with data from the Moves app) to see how exercise relates to your progress in losing weight and to your mood and satisfaction levels. We take great care to ensure the security of this information. All data will be assigned numerical codes and stored anonymously on our servers, so that it can be used in aggregate form for research purposes.*

*You are in control of the way that the app works. You can use the Settings page to determine which items you will record and how often (you can change these settings at any time). Use the Stats page to view your journey and look at your progress over time. You could choose to export this data for your own records – you could also show it to your GP or other medical specialist if you think it helps to communicate to them what you have been doing and how you are getting on.*

The app has a welcome page, a settings page, a journal page(s), and a statistics page. Each page is described below.

*Welcome* – This describes in simple terms what the app will do and how to use it.

*Settings* – This is where people can choose what to record and how often. Also when the app will prompt them.

*Journal* – This is where data is entered.

*Stats* – This is where the results are displayed s and enables the user to email a specific date range to a friend or to their GP or specialist.

Example screenshots of the app are provided below.


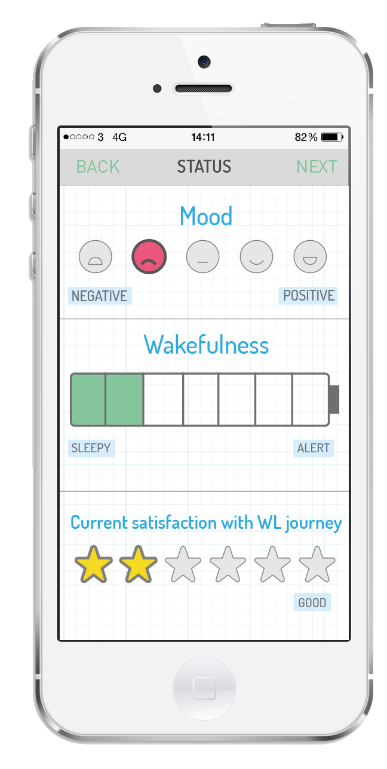

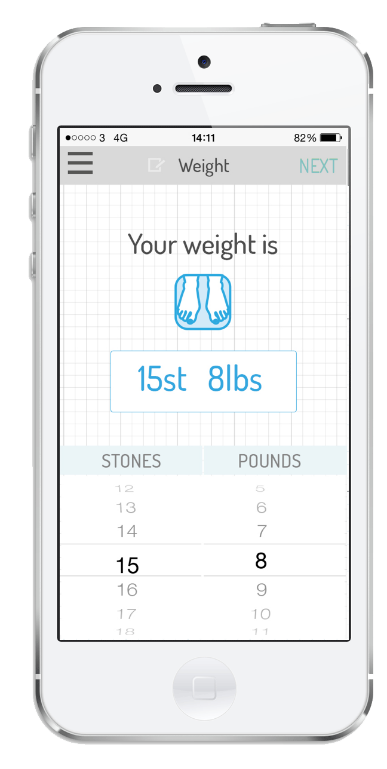


The App also shows the user their data on demand in a graphical format. It can display data items individually or all together as individual coloured lines so that they can be easily compared. Weekly physical activity can be calculated by adding the number of minutes spent in additional exercise (as entered into the journal) to the total weekly physical activity recorded automatically by the Moves App (which classifies movement as walking, cycling, running), ignoring time spent on transport or idle.

**Data Security**

Subjects’ physical activity can be tracked using the commercial application included in Moves by ProtoGeo that uploads data from their smartphones to the supplier's servers. When participants download and install the app, they can be directed to the supplier’s terms of service (<https://moves-app.com/fullterms> ) and to their privacy policy (<http://www.moves-app.com/privacy> ). The app records the following items: Location, Accelerometer data (the equivalent of a pedometer), Wi-Fi network IDs and activity data. It also collects other information about the phone’s operating system, device identifier, carrier, language, battery performance, wi-fi or other network connections, and other data that the user has permitted the App to access on their device (through permissions settings). The data is uploaded from their phone over a secured connection to the company’s servers that analyse the data and provide a running total of physical activity classified into walking, running, cycling and motorised transport. The results are sent back to the subject’s phone over a secured connection so that they can view and interact with them. The results are also stored on the company’s servers. If the user labels a place or changes an activity type, the information is also collected and stored in the company’s database. The company, Protogeo, use commercially reasonable physical and technical safeguards to secure the user’s data and encrypt the communication channels. If the user chooses to communicate directly with ProtoGeo their database (which may be located outside of the EU) can include personally identifying information.

The sequence of events is as follows: User visits our website and obtains the links to download the Moves and WLCompanion Apps. When the WLCompanion app first runs it requests the user’s research participant trial number and date of birth as an anonymous identifier. It also checks whether Moves app has been downloaded and prompts the user to do so if it hasn’t. When the Moves app is also downloaded, the WLCompanion app automatically requests access to the Moves data, which the user can choose to grant or decline.

ProtoGeo provides a secure method for the research team to retrieve a subject’s data (excluding their personally identifying information such as name, email address or phone number) provided that the subject has given their permission for the research to do so. This is provided in a secure way by means of a secret keycode that a research team’s software can associate with the research participants’ trial number, hence all data can be maintained in an anonymised form. The participants’ physical activity data can be transmitted to a research team’s servers over a secured connection in encrypted form.

All data from the WLcompanion app can also be stored by the research team without any personal data such as name or address in encrypted format on the research team’s server, using the research participant’s trial number as an anonymous identifier.
