# Supplementary material for: Tracking physical activity using smart phone apps: assessing the ability of a current app and systematically collecting patient recommendations for future development
Source: BMC Med Inform Decis Mak. 2020 Feb 3;20:17. doi: 10.1186/s12911-020-1025-3 (PMC6998214; doi:10.1186/s12911-020-1025-3)
Supplement: Supplementary file 2 — Additional file 2. Theoretical Domains Framework Survey Items [file 12911_2020_1025_MOESM2_ESM.docx]

**Additional File 2.**

Theoretical Domains Framework Survey Items

| Theoretical Domain | Item | Response Options  {all presented on 5-point scales} |
| --- | --- | --- |
| Knowledge | WLcompanion enables me to record my weight each week. | {1, Strongly Disagree; 2, Disagree; 3 neither disagree or agree; 4, agree; 5 strongly agree} |
|  | WLcompanion can graph my physical activity and weight. | {1, Strongly Disagree}... |
|  | WLcompanion can help me keep track of my progress after surgery. | {1, Strongly Disagree}... |
|  | Walking at least the following amount is good for your health | {1, 10 min per day}... |
|  | I am aware of apps for monitoring my physical activities (such as walking), weight or mood. | {1, Strongly Disagree}... |
|  | The Moves app can show me how many minutes and steps I’ve walked every day provided I carry my phone with me. | {1, Strongly Disagree}... |
|  | The Moves app can show me a daily account of my physical activity and places I've visited. | {1, Strongly Disagree}... |
|  | I often visit the website [www.wlcompanion.com](http://www.wlcompanion.com). | {1, Strongly Disagree}... |
| Reinforcement | I use these apps without having to think about them. | {1, Strongly Disagree}... |
|  | I respond to the phone's reminder to record my weight weekly. | {1, Strongly Disagree}... |
|  | Using Moves I can track my progress without having to think about it. | {1, Strongly Disagree}... |
| Environmental Context and Resources | I take my phone everywhere I go. | {1, Strongly Disagree}... |
|  | I use an electronic watch or wristband to track my activity. | {1, Strongly Disagree}... |
|  | I use an app to track what I eat or my calorie intake. | {1, Strongly Disagree}... |
|  | I am not allowed my mobile phone at work. (reverse scored) | {1, Strongly Agree}... |
|  | I turn off some services on my phone in order to preserve battery life. (reverse scored) | {1, Strongly Agree}... |
|  | My phone's battery runs out of power during the day. (reverse scored) | {1, Strongly Agree}... |
|  | I put my phone down when I am at home. (reverse scored) | {1, Strongly Agree}... |
|  | I always keep my phone charged. | {1, Strongly Disagree}... |
|  | My work prevents me from taking more exercise (e.g. shift work (reverse scored) | {1, Strongly Agree}... |
|  | I would walk more if there were better facilities (e.g. parks near my home). (reverse scored) | {1, Strongly Agree}... |
|  | Childcare commitments prevent me from taking more exercise. (reverse scored) | {1, Strongly Agree}... |
| Memory Attention and Decision Processes | When I am at work, I usually carry my phone around with me | {1, Strongly Disagree}... |
|  | I forget to take my phone with me when I leave the room. (reverse scored) | {1, Strongly Agree}... |
|  | I weigh myself regularly. | {1, Strongly Disagree}... |
|  | I usually carry my phone around with me at home or when I go out. | {1, Strongly Disagree}... |
|  | My phone is usually in my handbag or on the desk when I am at work. (reverse scored) | {1, Strongly Agree}... |
| Behavioral Regulation | I like to increase my activity goal from week to week. | {1, Strongly Disagree}... |
|  | I set a goal for my weekly activity. | {1, Strongly Disagree}... |
|  | I check my weekly activity against my goal. | {1, Strongly Disagree}... |
|  | I take opportunities for extra physical activity as they arise. | {1, Strongly Disagree}... |
|  | I keep track of my health improvement progress. | {1, Strongly Disagree}... |
| Intentions | After my surgery I intend to walk more than I do now | {1, Strongly Disagree}... |
|  | After my surgery, I will use these apps to track my weight | {1, Strongly Disagree}... |
|  | After my surgery, I will use these apps to track my physical activity | {1, Strongly Disagree}... |
| Social Influences | Some people I know use a wristband or an app to track their exercise. | {1, Strongly Disagree}... |
|  | People I know offer encouragement when they see me using an app to track my weight and activity. | {1, Strongly Disagree}... |
|  | I would recommend my friends to use Moves. | {1, Strongly Disagree}... |
| Social and Professional Roles and Identity | People who are overweight need to take every opportunity for more exercise. | {1, Strongly Disagree}... |
|  | Apps like these are useful for people who have bariatric surgery. | {1, Strongly Disagree}... |
|  | Using the WLcompanion app contributes to research that will help overweight people in the future. | {1, Strongly Disagree}... |
| Beliefs about Consequences | I can improve my health by using Moves and WLcompanion. | {1, Strongly Disagree}... |
|  | Health monitoring apps will help me improve my lifestyle. | {1, Strongly Disagree}... |
|  | Increasing my physical activity will improve my health. | {1, Strongly Disagree}... |
| Beliefs about Capabilities | I would walk more if it was easier (e.g. if you have arthritis or other mobility problems). (reverse scored) | {1, Strongly Agree}... |
|  | I think it would be too difficult to use both Moves and WLcompanion all the time. (reverse scored) | {1, Strongly Agree}... |
|  | I get breathless when I walk. reverse scored | {1, Strongly Agree}... |
| Skills | I find it easy to enter my information into WLcompanion. | {1, Strongly Disagree}... |
|  | I know how to install new apps on my Smartphone and regularly do so. | {1, Strongly Disagree}... |
|  | I regularly install new apps on my phone. | {1, Strongly Disagree}... |
|  | I use apps to track my diet, weight or physical activities. | {1, Strongly Disagree}... |
| Goals | Generally, other things are more important than ensuring I use these apps. (reverse scored) | {1, Strongly Agree}... |
|  | Increasing my physical activity is a very important goal for me. | {1, Strongly Disagree}... |
|  | I can generally find something better to do with my time than extra walking. (reverse scored) | {1, Strongly Agree}... |
| Emotions | I enjoy seeing my physical activity trending upward. | {1, Strongly Disagree}... |
|  | I get a sense of satisfaction from recording my weight and reviewing my progress. | {1, Strongly Disagree}... |
|  | It is a burden to use these apps. | {1, Strongly Disagree}... |
|  | I generally feel worried or concerned about recording my physical activity. | {1, Strongly Disagree}... |
|  | I generally feel unhappy or depressed. | {1, Strongly Disagree}... |
|  | I generally feel good when I view my progress using the apps. | {1, Strongly Disagree}... |
|  | My weight has increased while I have been recording it with WLcompanion. | {1, Strongly Disagree}... |
|  | I dread weighing myself. | {1, Strongly Disagree}... |
|  | I am discouraged when I see a low physical activity report on Moves. | {1, Strongly Disagree}... |
